# Phytochromes in *Agrobacterium fabrum*

**DOI:** 10.3389/fpls.2021.642801

**Published:** 2021-04-29

**Authors:** Tilman Lamparter, Peng Xue, Afaf Elkurdi, Gero Kaeser, Luisa Sauthof, Patrick Scheerer, Norbert Krauß

**Affiliations:** ^1^Botanical Institute, Karlsruhe Institute of Technology KIT, Karlsruhe, Germany; ^2^Charité – Universitätsmedizin Berlin, Corporate Member of Freie Universität Berlin and Humboldt-Universität zu Berlin, Institute of Medical Physics and Biophysics, Group Protein X-ray Crystallography and Signal Transduction, Berlin, Germany

**Keywords:** crystal structure, protein conformational changes, plant infection, bacterial conjugation, light regulation, histidine kinase, evolution, origin of plant phytochromes

## Abstract

The focus of this review is on the phytochromes Agp1 and Agp2 of *Agrobacterium fabrum*. These are involved in regulation of conjugation, gene transfer into plants, and other effects. Since crystal structures of both phytochromes are known, the phytochrome system of *A. fabrum* provides a tool for following the entire signal transduction cascade starting from light induced conformational changes to protein interaction and the triggering of DNA transfer processes.

## Overview

Plant phytochromes and bacterial phytochromes are different but have many common features. Whereas plant phytochromes have been discovered by physiology and spectral assays (Butler et al., 1959), bacterial phytochromes were identified by their gene sequence, with the consequence that often their biological functions are unknown. Properties of bacterial phytochromes have been summarized after their discoveries and also more recently (e.g., Vierstra and Davis, 2000; Takala et al., 2020). The present review concentrates on *Agrobacterium fabrum* C58 or Agrobacterium tumefaciens C58 (synonyms for the same species) phytochromes which are investigated in our group. *A. fabrum* has two phytochromes termed Agp1 and Agp2 or AtBphP1 and AtBphP2. Both phytochromes may be regarded as model phytochromes of their own kind. In The Discovery of *A. fabrum* Phytochromes section, we will briefly describe the discovery of these phytochromes in the context of the discovery of other phytochromes. In Overall Distribution of Phytochromes section, we address the overall distribution of phytochromes and different domain organizations. Both Agp1 and Agp2 have been used for a number of biophysical studies and crystal structure analyses. These studies will be summarized in Light Induced Protein Conformational Changes and Protein Structure section, with the focus on protein conformational changes. Biological functions Agrobacterium phytochromes were discovered after the proteins have been analyzed in their isolated forms. We will accordingly describe the biological functions of these phytochromes in Multiple Biological Functions section. All in all, many steps in the phytochrome signal transduction, starting from light absorption, chromophore isomerization, protein conformational changes, signal transmission, and biological function are known from *A. fabrum*. Since the focus of the present issue is on plant phytochromes, we will also try to make links between *A. fabrum* and plants where appropriate.

### The Discovery of *Agrobacterium fabrum* Phytochromes

**Figure 1:**
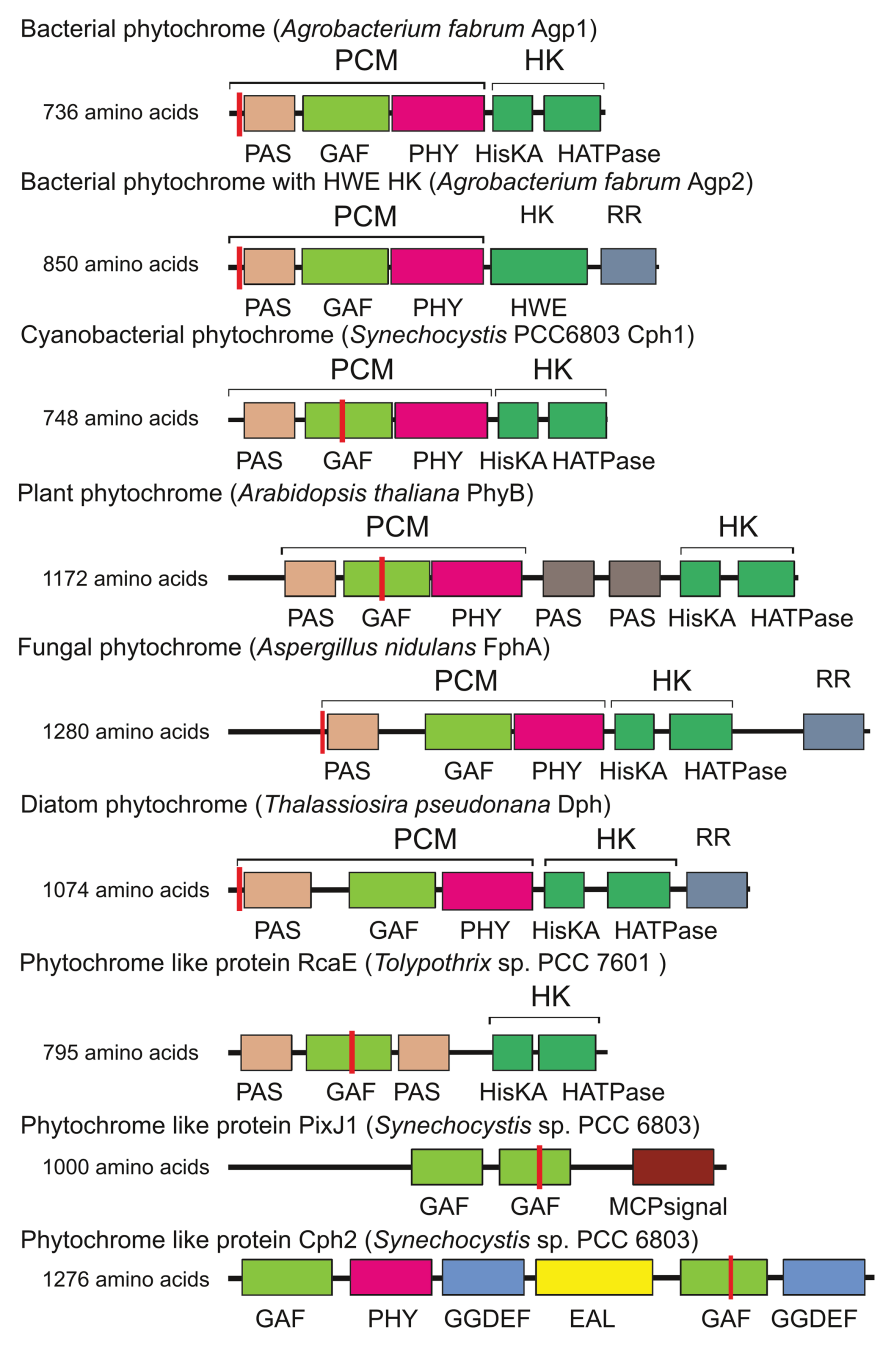
Domain arrangements of phytochromes and phytochrome-like proteins. PixJ, RcaE, and Cph2 are termed phytochrome-like proteins or cyanobacteriochromes here. Sites of covalent chromophore attachment are indicated by red vertical lines. HK, histidine kinase; RR, response regulator. Other abbreviations are domain names.

The first identified phytochrome sequence is from oats (Hershey et al., 1987), and other plant and green algal phytochrome sequences followed. The early impression was that phytochromes are restricted to plants and green algae, because no phytochrome effects were found in organisms outside plants and green algae (with a fungal exception; Valadon et al., 1979). Mutant studies revealed the first phytochrome-like protein in the cyanobacterium Fremyella diplosiphon (Kehoe and Grossman, 1996) and genome sequencing detected the first prokaryotic phytochrome Cph1 in the cyanobacterium Synechocystis PCC 6803 (Hughes et al., 1997; Lamparter et al., 1997; Yeh et al., 1997). The impression that prokaryotic phytochromes could be restricted to cyanobacteria was soon rejected because ongoing genome sequencing of other bacteria revealed these photoreceptors in species like *Deinococcus radiodurans* (Davis et al., 1999), Bradyrhizobium sp., *Rhodopseudomonas palustris* (Giraud et al., 2002), or *Pseudomonas aeruginosa* (Tasler et al., 2005). The discovery of *A. fabrum* phytochromes also followed genome sequencing. In our group, the sequences of both phytochromes were found in a BLAST search just before the two publications of the genome came out (Goodner et al., 2001; Wood et al., 2001). For plant biologists, *A. fabrum* is an interesting bacterium in several aspects. We, therefore, wanted to include the phytochrome system of *A. fabrum* in ongoing studies. We first cloned the phytochromes Agp1 and Agp2 into expression vectors and started with studies on recombinant protein with the more conventional Agp1. Expression and purification of Agp1 and Agp2 was easier as compared to Cph1 (Lamparter et al., 1997), with which we had worked before. The group of Vierstra also studied Agp1 and Agp2 (they termed the proteins AtBphP1 and AtBphP2). Their studies revealed the bathy-phytochrome character of Agp2 (Karniol and Vierstra, 2003). As for most bacterial phytochromes, the discovery of these phytochromes was not connected to a biological function. No light effect was described for *A. fabrum*. Therefore, knockout mutants were required to unravel phytochrome effects. In *A. fabrum*, *agp1*^−^ and *agp2*^−^ mutants and *agp1*^−^/*agp2*^−^ double mutants were generated quite early (Oberpichler et al., 2006), but a clear mutant phenotype was not found. Our group continued to study the recombinant proteins, which indeed could be produced in large amounts that allowed broad biochemical characterization including crystallization. We discovered that the position of the amino acid residue involved in covalent attachment of the chromophore is located just N-terminal of the PAS domain. This position is different from that of plant and cyanobacterial phytochromes (see also Figure 1). We initially found that the chromophore is bound covalently to a cysteine, as in plant phytochromes, but the homologous cysteine of plant phytochromes is missing in Agp1. We mutated all three cysteines of Agp1 and showed that the cysteine at position 20 is the most likely candidate (Lamparter et al., 2002). This finding was confirmed by mass spectrometry on proteolytic Agp1 fragments (Lamparter et al., 2004). This chromophore binding site is used by most bacterial, fungal, and heterokont phytochromes.

### Overall Distribution of Phytochromes

Here, we want to put *A. fabrum* phytochromes into an overall context, regarding their homologies, domain structures, and chromophores. Phytochromes are present in all land plants including mosses, ferns, and angiosperms, in many green algae, in fungi, diatoms, and in brown algae (Mooney and Yager, 1990; Kendrick and Kronenberg, 1994; Schneider-Poetsch et al., 1994; Blumenstein et al., 2005; Froehlich et al., 2005; Fortunato et al., 2016) but are missing in major groups such as red algae, archaea, and animals. In a large number of bacterial species, phytochromes were found, but not all bacteria have a phytochrome (Lamparter, 2006). Phytochromes are also present in the slime mold *Physarum polycephalum* (Lamparter and Marwan, 2001; Schaap et al., 2016). Slime molds are the phylogenetic sister group of ophistokonts (animals, fungi, amoebae, and others).

**Figure 2:**
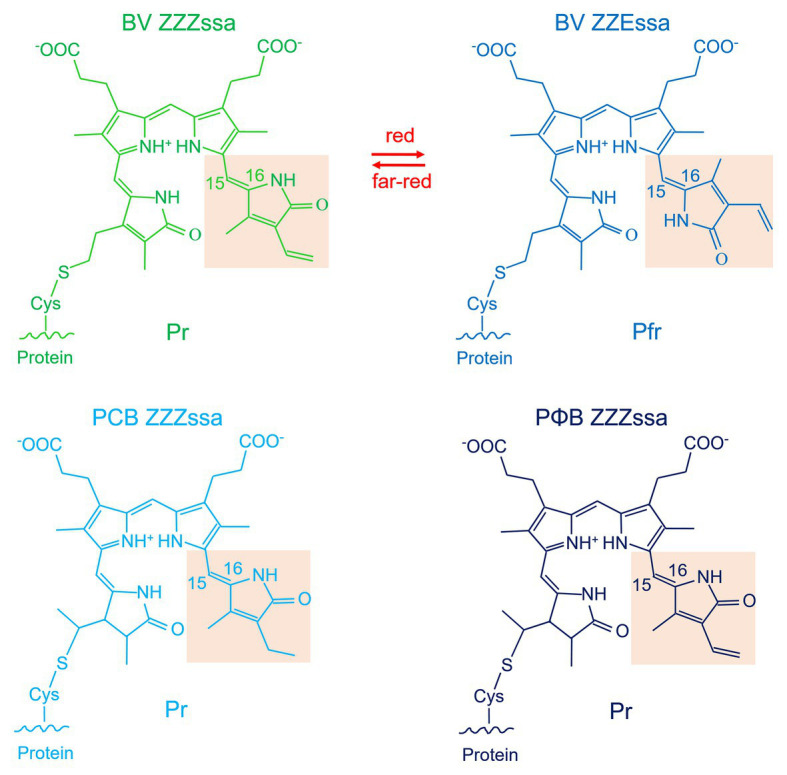
Chromophores of phytochromes. Shown are the structures that result from covalent attachment to the respective conserved cysteine residue. BV is depicted in the *ZZZ* and *ZZE* configurations that refer to Pr and Pfr, respectively. Note that the spectral properties of Pr and Pfr are not only determined by the isomerization states of the double bonds but also by the interaction with the amino acids of the chromophore pocket. The BV double bond arrangement around ring A is drawn according to the Agp1-PCM-SER13 structure (PDB entry 5HSQ; Nagano et al., 2016), in other structures (e.g., Wagner et al., 2005), the double bond is modeled outside the ring like in free phytochromobilin.

A typical phytochrome consists of a PAS domain (Aravind and Ponting, 1999), a GAF domain (Aravind and Ponting, 1997), and a PHY domain, which are combined to become the N-terminal photochromic core module (PCM) with the covalently bound linear tetrapyrrole (bilin); the C-terminal part is variable, but most often a histidine kinase module (Hiskin). Histidine kinases act together with response regulators (RR) in so-called two component systems (Gao and Stock, 2009). The phytochrome domain arrangements are given in Figure 1 (Rockwell et al., 2006; Buchberger and Lamparter, 2015; Lamparter et al., 2017). Here, we refer to a typical phytochrome only if the entire PAS-GAF-PHY tri-domain is present. The domain arrangements of fungal and plant phytochromes are PCM-Hiskin-RR and PCM-PAS-PAS-Hiskin, respectively. Agp1 and many other bacterial phytochromes have the domain arrangement PCM-Hiskin, but Agp2 and several other bacterial phytochromes of rhizobiales have the unusual domain arrangement of PCM – Hiskin – RR (Figure 1). The chromophore in bacteria and fungi is biliverdin (BV; Lamparter et al., 1997; Bhoo et al., 2001; Blumenstein et al., 2005; Froehlich et al., 2005), which is formed from heme by the enzyme heme oxygenase. Phytochromobilin (PΦB), the chromophore of plant phytochromes (Lagarias and Rapoport, 1980; Rüdiger and Thümmler, 1994), is synthesized in a one-step reaction from BV by the enzyme PΦB synthase (Kohchi et al., 2001; Chemical structures of chromophores are given in Figure 2). The phytochromes of heterokont algae (encompassing diatoms and brown algae) have also BV as a chromophore (Fortunato et al., 2016). Cyanobacterial phytochromes have either BV or phycocyanobilin (PCB) as chromophore, the latter being synthesized by a single enzyme, PCB synthase, which catalyzes two subsequent reduction steps (Frankenberg et al., 2001). As noted above, PΦB or PCB chromophores are covalently bound to a cysteine in the GAF domain of the PCM of plant or cyanobacterial phytochromes (Figure 1; Lagarias and Rapoport, 1980; Rüdiger and Thümmler, 1994), respectively, whereas BV is bound to a conserved cysteine N-terminal of the PAS domain of PCM of bacterial or fungal phytochromes (Figure 1; Lamparter et al., 2002; Lamparter, 2004). The common feature among phytochromes is their photoconversion between two conformational states, the Pr (red absorbing) and the Pfr (far-red absorbing) forms, which is triggered by an isomerization of the bilin chromophore (Figure 2). The maxima of Pr forms extend from 655 nm for cyanobacterial Cph1 to 665 nm for plant phytochromes to about 700 nm for biliverdin binding bacterial or fungal phytochromes. The maxima of Pfr forms are shifted by 45–65 nm to longer wavelengths.

As has been reported for many protein families with a modular architecture, a variable domain arrangement can be seen for phytochromes and related proteins in different organisms. Despite the many different domain combinations, the PCM arrangement (PAS-GAF-PHY) is found in bacteria, plants, and fungi (Lamparter et al., 2017). In cyanobacteria, many photoreceptor proteins have been found that have a bilin-binding GAF domain combined with other domains (de Marsac and Cohen-Bazire, 1977; Kehoe and Grossman, 1996; Yoshihara et al., 2000, 2004; Wilde et al., 2002; Ng et al., 2003; Terauchi et al., 2004; Kehoe and Gutu, 2006; Moon et al., 2011; Hirose et al., 2013); the domain arrangements of some examples are given in Figure 1. These proteins are often termed cyanobacteriochromes or phytochrome-like proteins, sometimes knotless (see below) phytochromes.

**Figure 3:**
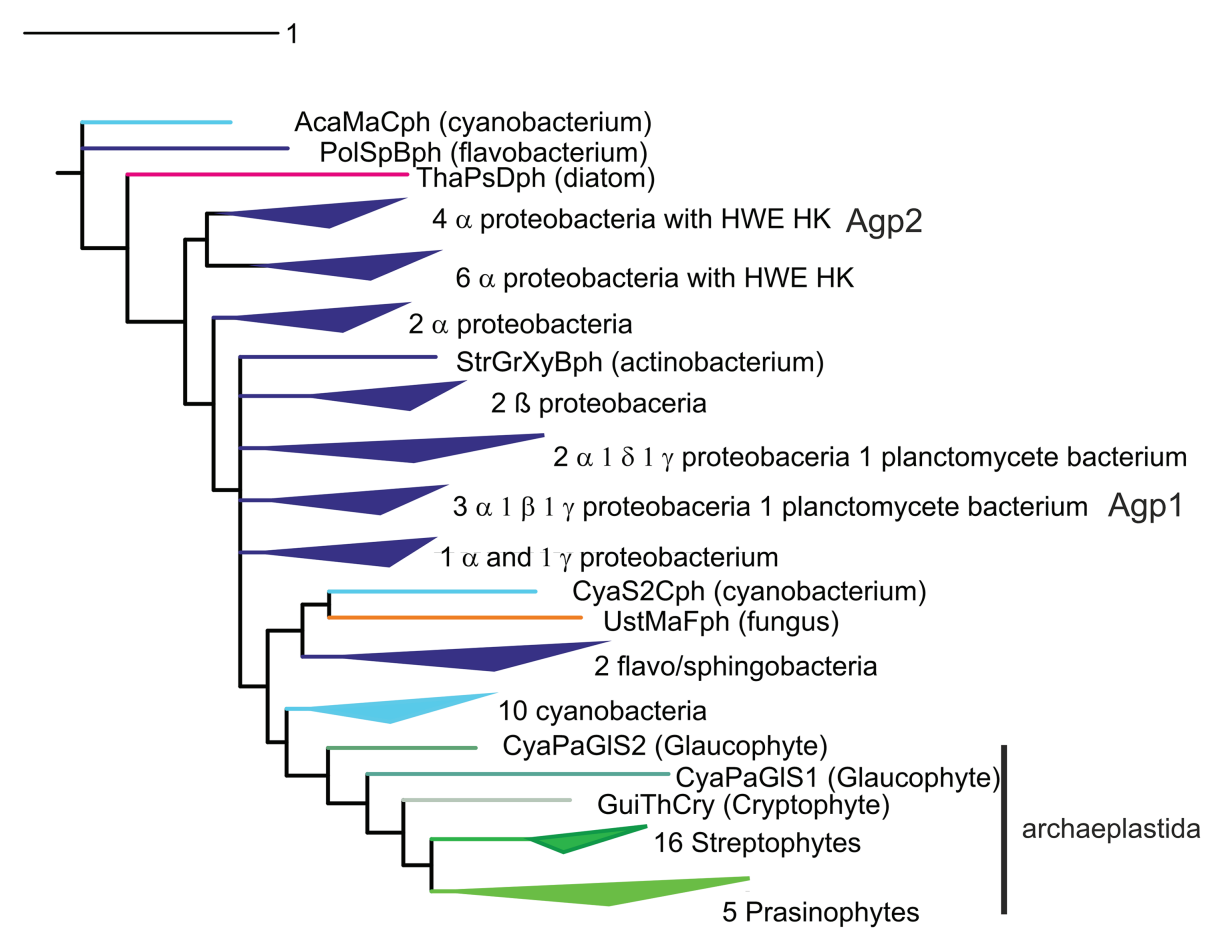
Phylogenetic tree of phytochromes, based on a Muscle alignment (Madeira et al., 2019) of photochromic core module (PCM) sequences and the phylogeny program Mr. Bayes (Ronquist et al., 2012), adapted from Kooss and Lamparter (2017).

Phylogenetic relationships between phytochromes are often difficult to resolve. Among bacteria, the patterns of phytochrome phylogeny do not necessarily follow the relationships among bacteria. Agp1 and Agp2 (Lamparter et al., 2002, 2017; Karniol and Vierstra, 2003) appear always in different clades in phylogenetic trees (as in Figure 3). Such findings can be explained by horizontal gene transfer through conjugation or natural transformation. On the other hand, other rhizobial phytochromes are found in a common clade (Rottwinkel et al., 2010), and cyanobacterial phytochromes form a separate clade as well (Lamparter, 2004). Within cyanobacteria, but also among other groups of bacteria, a phylogenetic analysis of the histidine kinase modules of phytochromes reveals different relationships than a tree formed by the photosensory core module (PCM) tri-domains. This argues for domain exchanges of one histidine kinase against another (Buchberger and Lamparter, 2015). The histidine kinase-like domain of plant phytochromes (Figure 1) could also have a different origin than the PCM. A single recombination could explain the domain pattern of plant phytochromes with two PAS domains in the center of the protein and a new histidine kinase (Lamparter, 2004; Buchberger and Lamparter, 2015). Phytochromes evolved most likely before the cyanobacteria arose. As noted above, the BV chromophore in bacteria is produced from heme in a one-step enzymatic reaction, whereas the PCB chromophore of cyanobacterial phytochromes (Jorissen et al., 2002) is formed from BV in another enzymatic reaction (Frankenberg et al., 2001). The two-step synthesis of the cyanobacterial chromophore from heme can be considered as an advanced feature, meaning that phytochromes with a PCB chromophore have most likely evolved from phytochromes with a BV chromophore, and not vice versa. The fact that phytochromes are broadly distributed in bacteria and that eukaryotes like fungi, heterokonts and slime molds also have phytochromes (although there is no direct evolutionary link between cyanobacteria and these groups) also suggests that phytochromes evolved before the appearance of cyanobacteria. The tri-domain arrangement could have been formed in several steps: A biliverdin binding GAF domain (non-covalent binding) could have evolved into a photosensory module. Then, a fusion with a PAS domain could have led to covalent binding of the chromophore. Finally, a fusion with a PHY domain could then have led to phytochrome-specific signal transduction, such as regulation of histidine kinase. The cyanobacteriochromes, in which a PCB chromophore is covalently bound to a GAF domain, but which are lacking the PAS and/or PHY domain, apparently evolved within cyanobacteria. The most likely scenario for the phytochrome evolution in cyanobacteria is that (i) already at the beginning of their evolution, cyanobacteria had phytochromes with a BV chromophore, inherited from their bacterial ancestors, (ii) the invention of PCB as antenna chromophore of phycobiliproteins led to a switch of the chromophore in most cyanobacterial phytochromes and to a switch of the binding site in the GAF domain, and (iii) with a chromophore binding site in the GAF domain, photoconversion became independent from the PAS domain, a prerequisite for domain rearrangement and for the formation of cyanobacteriochromes.

There are several scenarios how phytochromes could have entered the eukaryotic domain. Plants and green algae together always form a clear clade in phylogenetic trees of phytochromes, completed by glaucophytes and other members of the archaeplastida, pointing to a common origin of these phytochromes. However, it is difficult to understand phylogenetic trees with respect to the origin of the archaeplastidal lineage. Could it be that archaeplastidal phytochromes are derived from the cyanobacterial endosymbiont that gave rise to plant plastids? The variability of phytochrome sequences is rather high and it is difficult to obtain clear branch points for diversifications that are dated billions of years back. In published trees, the plant or archaeplastidal clade originates always at different positions in the prokaryotic domain, usually next to proteobacteria (compare, e.g., Lamparter, 2004; Rottwinkel et al., 2010; Duanmu et al., 2014; Buchberger and Lamparter, 2015; Fortunato et al., 2016). Hence, the hypothesis of cyanobacterial origin of archaeplastidal phytochromes is usually discarded (Duanmu et al., 2014). However, in some of our studies, plant phytochromes appeared sometimes as sister clade of cyanobacteria (Buchberger and Lamparter, 2015; Kooss and Lamparter, 2017; Figure 3). This makes a cyanobacterial origin of plant phytochromes more likely. The cyanobacterial origin is strengthened by recent yet unpublished studies in our group in which we concentrated on the amino acids of the chromophore binding site.

**Figure 4:**
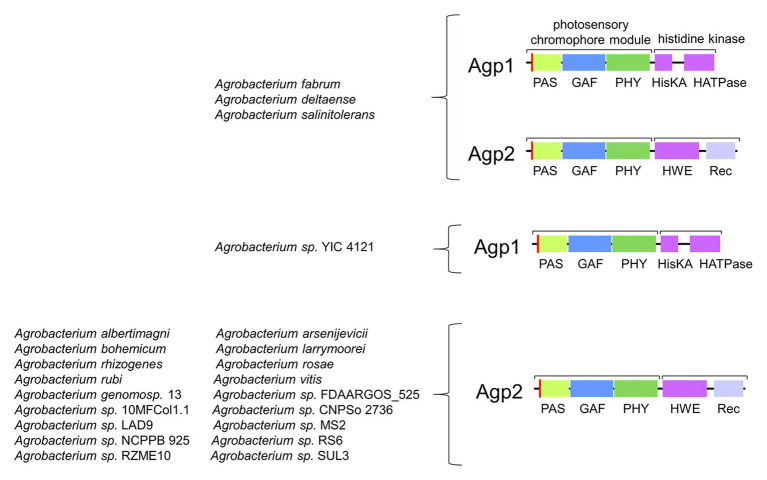
Abundance of Agp1 and Agp2 homologs in 22 *Agrobacterium* species.

Agp1 has a common domain arrangement of PAS-GAS-PHY-Hiskin (type 1 in Rottwinkel et al., 2010) and it is related to other rhizobial phytochromes with the same or similar type 4 domain arrangement (in Rottwinkel et al., 2010). Agp2 (type 2 domain arrangement) has an additional response regulator at its C-terminus and is related to other rhizobial phytochromes of the same type. The two *Agrobacterium* phytochromes are antagonistic with respect to their dark state. Agp1 has a Pr dark state, as almost all other phytochromes, whereas Agp2 has a Pfr dark state (Karniol and Vierstra, 2003), a feature which is only found for some related rhizobial type 2 proteins and single other phytochromes like *P. aeruginosa* BphP1 (Tasler et al., 2005) or Bradyrhizobium BphP1 (see below). These latter types of phytochromes with a Pfr dark state are now termed bathy phytochromes. In Pr and Pfr, the chromophore adopts a *ZZZ* and *ZZE* isomeric states, respectively, and in solution the configuration is *ZZZ* (Lamparter and Michael, 2005). Upon incorporation into the protein, the isomeric state is retained, which is probably the reason why most phytochromes have a Pr dark state. Consequently, the chromophore must undergo isomerization after incorporation into the protein in bathy phytochromes. a process that can easily be followed by spectrophotometry (Lamparter and Michael, 2005; Inomata et al., 2006, 2009). The two *A. fabrum* phytochromes together cover a broader spectral range in the long wavelength region of visible light or short wavelength infrared range than only one phytochrome would. Quite interestingly, out of 22 different *Agrobacterium* species, only three have both Agp1 and Agp2 homologs, one has an Agp1 homolog only and 17 have Agp2 homologs only. The presence of Agp2 homologs is thus much more important than the presence of two phytochromes or the presence of Apg1 homologs (Figure 4), although in *A. fabrum*, both proteins seem to be equally important.

### Light Induced Protein Conformational Changes and Protein Structure

The phytochrome fingerprint is its photoconversion between the Pr and Pfr states. This step is triggered by Z-E isomerization of the double bond within the C-D connecting methine bridge (Figure 2); the overall methine bridge configuration changes from *ZZZ* to *ZZE*. The protein environment holds the chromophore in such a way that only isomerization of the double bond within the methine bridge connecting rings C and D and the resulting rotation of ring D is possible. In the Pfr state, the protein is forced to keep the chromophore in a different geometry and local environment, so that the absorption maximum is red-shifted. It is thus essential that the phytochrome undergoes conformational changes during photoconversion within the chromophore pocket. In addition, the Pr/Pfr differential interaction between phytochromes and interaction partners such as PIF3 in plants (Ni et al., 1998) and the modulation of histidine kinase activity require further protein conformational changes. It is likely that conformational changes start in the chromophore pocket and propagate from there through the entire protein.

Due to the high yield of protein expression of Agp1 and Agp2, both proteins could well be used for biophysical and biochemical studies and for protein crystallization (Scheerer et al., 2006, 2010). Conformational changes of Agp1 were monitored and confirmed by size exclusion chromatography, limited proteolysis (Esteban et al., 2005; Noack et al., 2007), and analysis of phosphorylation. Autophosphorylation of Agp1 is strong in the Pr form and diminished to ca. 20% in the Pfr form. Agp1 adducts with chemically synthesized locked chromophores, in which the Pr or the Pfr form is arrested due to an additional carbon chain between ring C and D of the chromophore, show an autophosphorylation pattern comparable with the Pr and Pfr forms of the BV adduct (Inomata et al., 2005). The residual phosphorylation activity of Pfr is thus not (only) due to residual Pr present in the sample. The apoprotein shows a significantly stronger (~130%) autophosphorylation activity than Pr. The Pr/Pfr pattern is comparable with several other bacterial phytochromes such as Cph1 (Yeh et al., 1997). In our hands, there was no autophosphorylation of Agp2 wild type only mutants in which the Asp residue of the response regulator was replaced showed Pr/Pfr independent autophosphorylation (Xue et al., 2019). Interesting temperature effects – the kinase activity decreased with increasing temperature – were found for Agp1, leading to the suggestion that Agp1 could also act as a temperature sensor (Njimona and Lamparter, 2011; Njimona et al., 2014). Whereas Agp2 has its response regulator on the same protein as the histidine kinase, Agp1 has a cognate response regulator that is encoded by the same operon, which is transphosphorylated by Agp1. Unfortunately, the exact function of histidine kinase regulation by light is not known for any bacterial phytochrome system. In effects like flagella rotation or *A. fabrum* virulence, two component systems play a central regulatory role. For bacterial phytochromes, it must be considered that the histidine kinases play only a modulatory role, because kinase activity is often not light controlled and a target of the response regulator has not been found yet.

**Figure 5:**
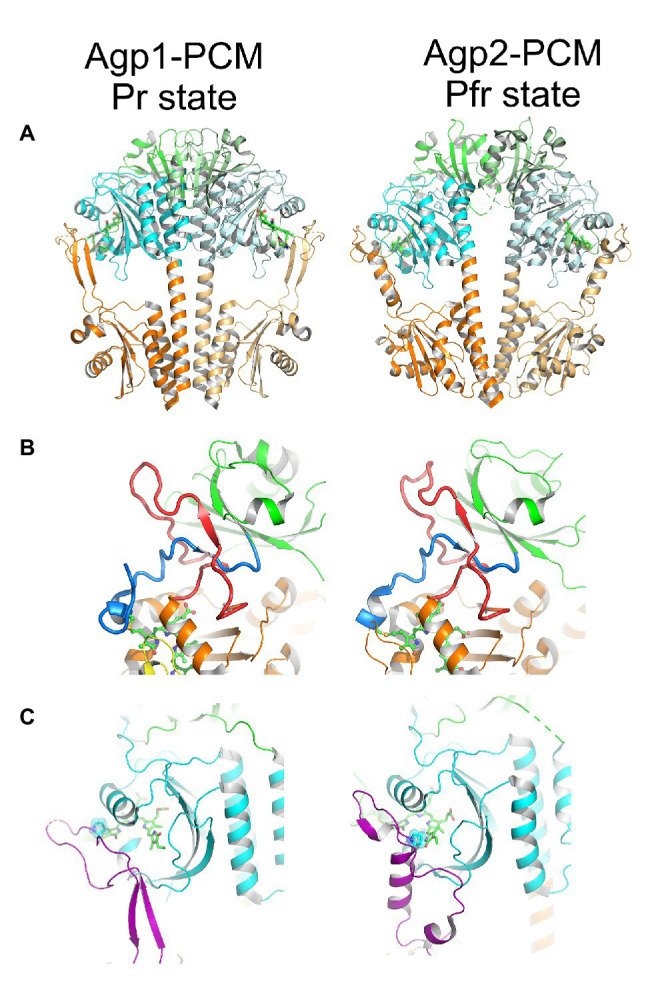
The structure of Agp1-PCM (left) in the Pr state and of Agp2-PCM (right) in the Pfr state. PCM, photosensory core module. Figures were constructed with PyMOL (The PyMOL Molecular Graphics System, Version 1.3 Schrödinger, LLC) using the protein data bank (PDB) entries 5I5L (Agp1-PCM) and 6G1Y (Agp2-PCM; Nagano et al., 2016; Schmidt et al., 2018). **(A)** Parallel crystallographic dimer of Agp1-PCM and non-crystallographic dimer of Agp2-PCM shown in cartoon representation, PAS domains in green, GAF domains in cyan, and PHY domains in orange. The biliverdin chromophores are shown in ball and stick representation. **(B)** The knotted structures formed between the N-terminal extensions (blue) of the PAS domains and the so-called lasso subdomains (red) within the GAF domains. **(C)** The tongue subdomains of the PHY domains, shown in purple, fold into different secondary structures in the Pr state as seen in Agp1-PCM and in the Pfr state as seen in Agp2-PCM. The proline residues of the conserved PRxSF motifs that interact with ring A and ring D of BV in Agp1 and Agp2, respectively, are highlighted. Please note that only the long helix in the tongue region of Agp2-PCM is an *α*-helix, whereas the two shorter helical segments are 3_10_-helices. Corresponding figures of Agp1-PCM and Agp2-PCM are shown in identical views after superpositioning the PAS-GAF bi-domains.

Details on protein conformational changes could be obtained from the structures of Agp1-PCM and Agp2-PCM. Since there are now more than 30 phytochrome structures (PDB entries 1ZTU, 2O9C, 2OOL, 2VEA, 3ZQ5, 3G6O, 3IBR, 3S7P, 3S7Q, 4IJG, 4GW9, 4E04, 4O0P,4O01,4OUR, 4XTQ, 5AKP, 5I5L, 5HSQ, 5K5B, 5LLW, 5LLX, 5LLY, 6ET7, 6G1Y, 6G1Z, 6G20, 6BAF, 6BAY, 6BAO, 6BAP, 6BAK, 6PU2, 6PTX, 6PTQ, 6TC5, 6TL4, and 6TC7), the Agp1 and Agp2 structures can be discussed in a general context. Three of the structures are from full length phytochromes (Bellini and Papiz, 2012; Otero et al., 2016; Gourinchas et al., 2017), but none of these has a histidine kinase domain, so that the modulation of kinase activity cannot be clearly addressed on the structural level yet. The Agp1-PCM structure is in the Pr form and the Agp2-PCM structure in the Pfr form. Both could, therefore, reflect the general conformational changes during photoconversion. The structure of *D. radiodurans* phytochrome was obtained in the Pr and Pfr forms (Burgie et al., 2014a,b; Takala et al., 2014). Our Agp1-PCM and Agp2–PCM structures are comparable with these Pr and Pfr structures, respectively, which justifies the above assumption. As an example, we show here the structure of Agp1-PCM (PDB entry 5I5L; Nagano et al., 2016) and Agp2-PCM (PDB entry 6G1Y; Schmidt et al., 2018) as representatives of Pr and Pfr, respectively (Figure 5).

The phytochrome chromophore is embedded in a binding pocket, which is formed by the GAF domain, but the PAS and the PHY domains also contribute *via* important interactions to the chromophore pocket. An exceptional knot is formed between the region just N-terminal of the PAS domain and the GAF domain (Figure 5B; Wagner et al., 2005). The knot contributes to the stability of the protein fold, but is apparently not required for photoreversibility, since cyanobacteriochromes that cannot form such a knot are also photoreversible proteins (Burgie et al., 2013). A subdomain within the PHY domain forms a tongue-like structure (Figure 5C) that folds back onto the chromophore pocket such that the proline residue of a conserved PRxSF motif gets in direct contact with the A and D rings of the bilin in the Pr state as in Agp1 and in the Pfr state as in Agp2, respectively (Nagano et al., 2016; Velázquez Escobar et al., 2017). In Agp1 and other typical Pr structures in general, the tongue contains in addition to an extended coiled region a two-stranded anti-parallel β-sheet, whereas in Pfr structures as in Agp2, the extended coiled region of the tongue is complemented by a single α-helix. Phytochromes, therefore, undergo secondary structure changes in the tongue during photoconversion. Since the PHY domain is linked to the output module, these changes could then be converted in some form into changes in the activity of the output module.

In the bathy phytochrome PaBphP (Yang et al., 2011), irradiation of Pfr crystals at different temperatures revealed insight into very early photoinduced structural changes of the chromophore in three Lumi-F states, i.e., the *E* → *Z* isomerization at the C15–C16 double bond of the methine bridge between the pyrrole rings C and D of BV.

Further conformational changes toward later Meta-F intermediates in the photocycle could be described for Agp2 (Schmidt et al., 2018). Crystals of wild-type Agp2-PCM (in the Pfr form) no longer diffracted after irradiation with far-red light, indicating conformational changes during Pr formation. However, crystals of a mutant of Agp2-PCM, termed Agp2-PAiRFP2, are stable upon irradiation, because it is trapped in the Meta-F states. Meta-F is an intermediate state formed during the Pfr-Pr conversion. During the subsequent Meta-F-Pr transition, further large conformational changes take place, which is why only the crystals of Agp2-PAiRFP2 remain intact upon irradiation (Schmidt et al., 2018; Kraskov et al., 2020).

**Figure 6:**
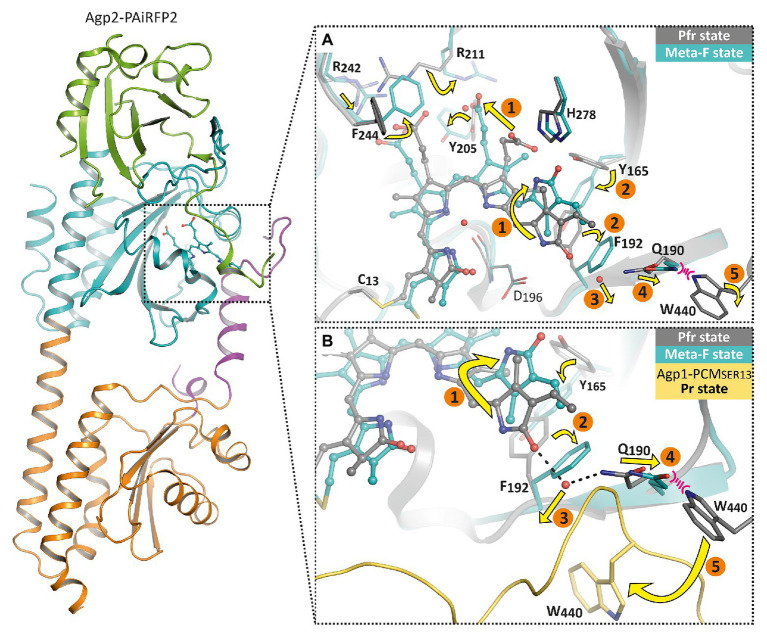
Overall fold and structural changes of the Agp2 variant Agp2-PAiRFP2 upon light absorption. The left panel shows the overall structure of Agp2-PAiRFP2 in the Meta-F state (PDB entry 6G20; Schmidt et al., 2018) in cartoon representation. The PAS domain is colored green, GAF domain in cyan, PHY domain in orange, and the PHY tongue is highlighted in purple. The right panels illustrate the proposed structural mechanism of the sequence of events for the Pfr to Pr transition by a superposition of the Pfr state (gray, PDB entry 6G1Z; Schmidt et al., 2018) and the Meta-F state (cyan) of Agp2-PAiRFP2, in which the chromophore is depicted as balls and sticks, highlighted amino acid residues as sticks and water molecules as red spheres. **(A)** The chromophore pocket is displayed. **(B)** A slightly changed view together with Agp1-PCM_SER13_ (yellow, PDB entry 5HSQ; Nagano et al., 2016) in the Pr state for comparison. After illumination, the chromophore isomerizes (1) accompanied by chromophore relaxation. Accordingly, the hydrogen bond network is changed and induces reorientations of Tyr165 and Phe192 (2). This affects the displacement of a water molecule (3). Thus, Gln190 shifts toward Trp440 (4). This steric clash could lead to an outward movement of Trp440 toward its position in Agp1-PCM_SER13_ (5) and also cause a refolding of the tongue region.

The crystal structures in the ground state and after irradiation showed that the overall folding of the protein remained unaffected, whereas chromophore surrounding amino acids rearrange accordingly. As a result of the crystal packing, the two monomers of Agp2-PAiRFP2 show two different Meta-F substates. These could represent two subsequent substates, so that a sequence of events of the first phytochrome activation steps could be formulated (1–5, Figure 6). These structural changes can essentially be divided into three groups. After illumination, the first group that undergoes changes is the chromophore itself and surrounding amino acids around both propionate side chains. For the Lumi-F state (like in PaBphP), the rotation of ring D (1, Figure 6) about almost 180° is visible. As a consequence of this rotation, the hydrogen bond network of ring D is altered; this is accompanied by a slight shift of His278. Afterward the chromophore relaxes due to changes in the propionate side chains (1, Figure 6). Thereby, Arg211 rotates strongly and interacts with the propionate side chain of ring C in the Meta-F state. Due to these positional changes, Phe244 (valine in wild-type Agp2) rotates directly above the propionate side chain of ring B and Arg242 shifts closer to the propionate side chain of ring B. In addition, Tyr205 rotates and interacts with the propionate side chains of ring B and C. The second group of structural changes occurs around ring D of the chromophore. The amino acids Tyr165 and Phe192 completely rearrange their orientation (2, Figure 6), whereby Phe192 displaces one water molecule that forms a hydrogen bond to ring D in Pfr (3, Figure 6). Immediately connected with this is a shift of Gln190 toward the highly conserved amino acid Trp440 (4, Figure 6). In this final process, a significant change in the orientation of the chromophore binding Cys13 can be observed. The position of Cys13 relative to the chromophore changes from one face to the opposite face. This movement is accompanied by a conformational transition of the N-terminal region. The third group describes the structural changes in the tongue of the PHY domain. As mentioned above, the displacement of Gln190 would cause a steric collision with Trp440. However, in the Meta-F crystal structure, Trp440 is located in a structurally unresolved region (disordered amino acids 439–448) of the tongue (5, Figure 6). This is an indicator of the first restructuring of the tongue toward a β-hairpin segment, which corresponds to the IR difference spectra between the final photoproduct of Agp2-PAiRFP2 and its dark state.

In summary, a complete chain of events from the chromophore to the tongue of Agp2 is obvious: the ring D isomerization of the chromophore triggers motion of Tyr165, which induces Phe192 rotation, which in turn causes a displacement of a water molecule and a shift of Gln190. From here, the signal moves to the tongue which can undergo secondary structure changes from α-helical to β-sheet. Other parts of the chromophore are also involved in positional changes of amino acids. How the signal moves from the tongue to the histidine kinase is a yet open question. The tongue is part of the PHY domain, which is connected by a long helix to the histidine kinase. Different mechanisms are possible for signal transmission from the PHY domain to the histidine kinase. An interesting possibility is raised by a comparison of Agp1-PCM crystal structures. There are altogether four crystal structures of Agp1-PCM in the PDB and the coordinates of seven symmetrically independent Agp1-PCM monomers (Nagano et al., 2016; 5I5L, 5HSQ, 6R26, and 6R27). These structures differ by an angle that is formed within the GAF-PHY connecting long helix. A detailed comparison of the structures shows that there is a hinge in the long helix around Met308. Amino acids of the tongue around Trp445 and Trp468 are also part of the hinge. This hinge allows flexibility of the PHY domain vs. the PAS and GAF domains of the PCM structure. This flexibility could hold for all phytochrome in the Pr form (see also Nagano et al., 2016 for discussion). For phytochrome structures in the Pfr state, there is no evidence for such a flexibility. On the contrary, all Pfr phytochrome structures have a straight long GAF-PHY connecting helix. The flexibility between the domains seems to be dependent on the form: flexible in the Pr and non-flexible in the Pfr form. Since the tongue is involved in the hinge, a photoconversion induced change in the tongue secondary structure could have a direct effect on this flexibility. The differential flexibility could have differential effects on the histidine kinase activity. The crystal structures are from the N-terminal tri-domain, the PCM; in the full length protein, two subunits are held together by the histidine kinase dimerization domain. PHY domains are thus restricted in their mobility. We assume that more subtle conformational changes in the PHY domain are transmitted to the histidine kinase.

### Multiple Biological Functions

In plants, many phytochrome responses such as the control of deetiolation (transition from pale to green appearance), seed germination or shade avoidance are related to photosynthesis (Kendrick and Kronenberg, 1994). Phytochrome control of chlorophyll synthesis, of photosynthesis proteins and of chloroplast maturation contributes directly to an efficient use of light energy: only when there is light and the plant can expect sufficient energy input, the plant spends the required energy for pigment and protein synthesis. Also, control of seed germination *via* phytochrome (Scheibe and Lang, 1969) results in maximum light capture for photosynthesis. By the shade avoidance response, a plant tries to overgrow other plants and maximize photosynthetic light capture (Smith and Whitelam, 1997). In non-cyanobacterial bacteria, diverse biological phytochrome responses were found. The first clear effect of a bacterial phytochrome was the regulation of bacteriochlorophyll synthesis and photosystem proteins in a photosynthetic bacterium, Bradyrhizobium, and in a related species, *Rhodopseudomonas palustris* (Giraud et al., 2002). One of the phytochromes in Bradyrhizobium, BphP1, is the first identified “bathy-phytochrome,” i.e., a phytochrome with a Pfr dark state. It was also shown that carotenoid synthesis is controlled by another phytochrome in Bradyrhizobium (Giraud et al., 2004), and phytochrome control of pigment synthesis was reported for the non-photosynthetic bacteria *D. radiodurans* and *P. aeruginosa* (Davis et al., 1999; Barkovits et al., 2011). Despite the regulatory role of phytochromes on photosynthetic of many organisms, in the cyanobacterium Synechocystis PCC6803, phytochrome Cph1 is not involved in the regulation of photosynthesis (Hübschmann et al., 2005).

**Figure 7:**
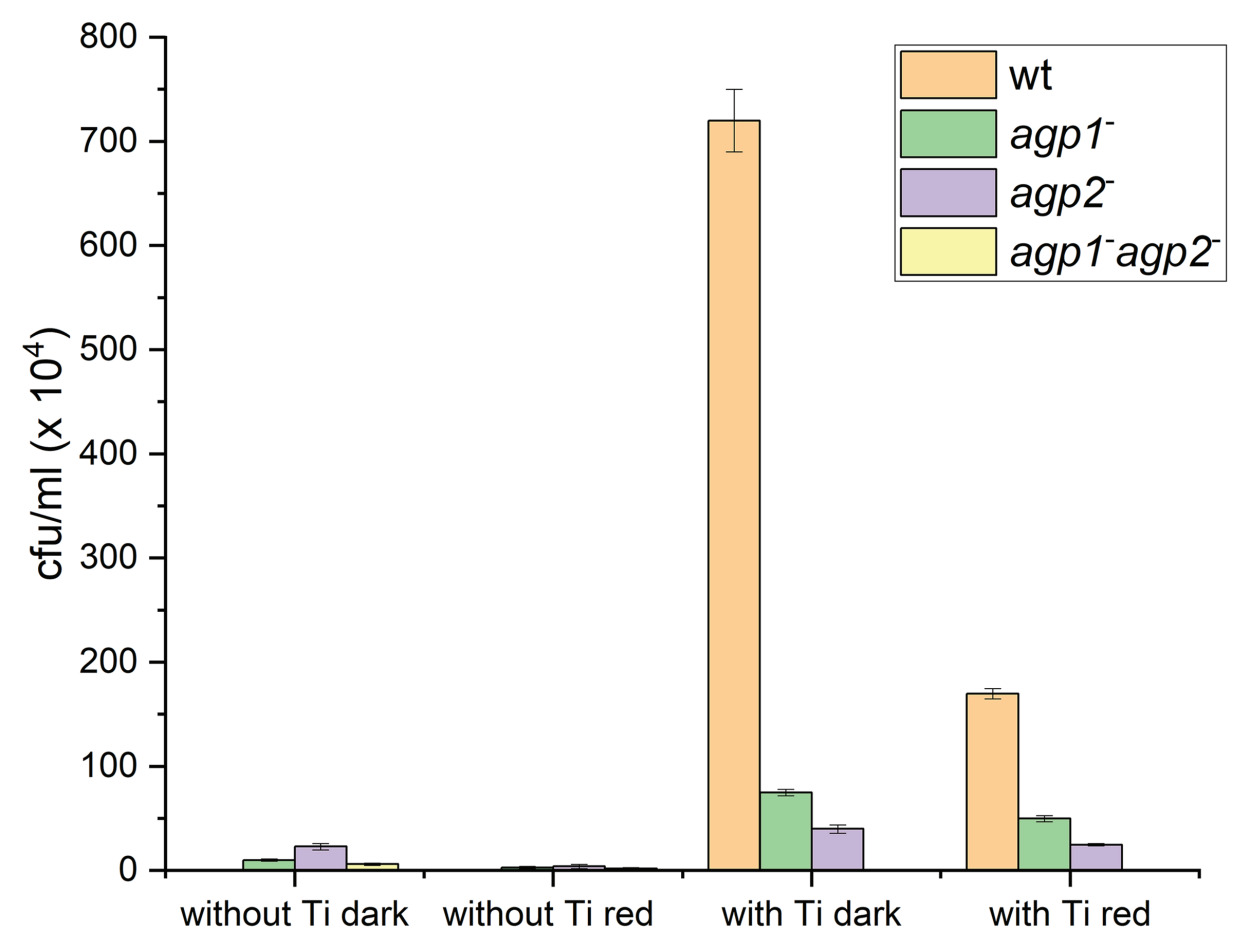
Conjugation in *Agrobacterium fabrum*. Transfer of the pBINGUS vector (with kanamycin resistance) from donor to recipient (ampicillin resistant) cells was monitored by kanamycin and ampicillin double resistance. Bar charts are based on data from (Bai et al., 2016). *A. fabrum* donor cells without or with Ti plasmid, mixed with recipient cells without Ti plasmid, were kept in darkness or treated with red light as indicated below the bars. The color code stands for the kind of donor strain used: wild type (orange), agp1^−^ mutant (green), agp2^−^ mutant (violet), or agp1^−^ /agp2^−^ (yellow). Z stands for conjugation rates of zero.

The biological functions of *Agrobacterium fabrum* phytochromes were first difficult to elucidate. Agrobacterium infects plants and induces the formation of plant tumors by DNA transfer. We tested the effect of light and phytochromes on plant infection, but initial tests were inconclusive. A first effect was discovered by a combination of a computer study and an experimental approach. In the computer study, it was found that TraA, a conjugation protein, has homologs in almost the same subset of Rhizobiales species as phytochromes (Lamparter, 2006; Bai et al., 2016). This pointed to a common function of TraA and phytochromes. Experimental conjugation studies pointed to a complex connection between Agp1, Agp2, and the conjugation machinery. *A. fabrum* has three TraA proteins, encoded by the Ti-plasmid, by the AT plasmid and by its linear chromosome (Lang et al., 2013). Each TraA has a mobA-mobL domain for DNA nicking, an ATPase domain, and a C-terminal helicase domain for DNA unwinding. All three TraA conjugal transfer proteins are larger than 1,000 amino acids. For donor and recipient cells, wild type and three different phytochrome knockouts have been used, and cells were prepared with or without Ti-plasmid (*A. fabrum* loses the Ti plasmid by prolonged growth at 37°C.) A strain without Ti-plasmid is expected to express two of three TraA proteins, although expression levels are presently unclear. Conjugation rates of wild-type or phytochrome mutants with and without Ti plasmid in donor cells are shown in Figure 7 (Bai et al., 2016). These data show that without Ti plasmid, conjugation was very low or zero. Wild-type donor cells without Ti plasmid did not induce any conjugation, whereas phytochrome mutants achieved low but significant conjugation levels. Red light reduced the conjugation in the single mutants. When donor cells with Ti plasmid were used, high conjugation rates were achieved in dark conditions, indicating a major role of Ti encoded TraA in conjugation. The rates of conjugation were much lower when cells were irradiated with red light or when agp1^−^ or agp2^−^ knockout mutants were used as donor cells. The agp1^−^/agp2^−^ double knockout donor induced no conjugation at all. The conjugation of *A. fabrum* is thus under the control of light and phytochromes. Whether or not the Ti plasmid is present makes a major difference.

**Figure 8:**
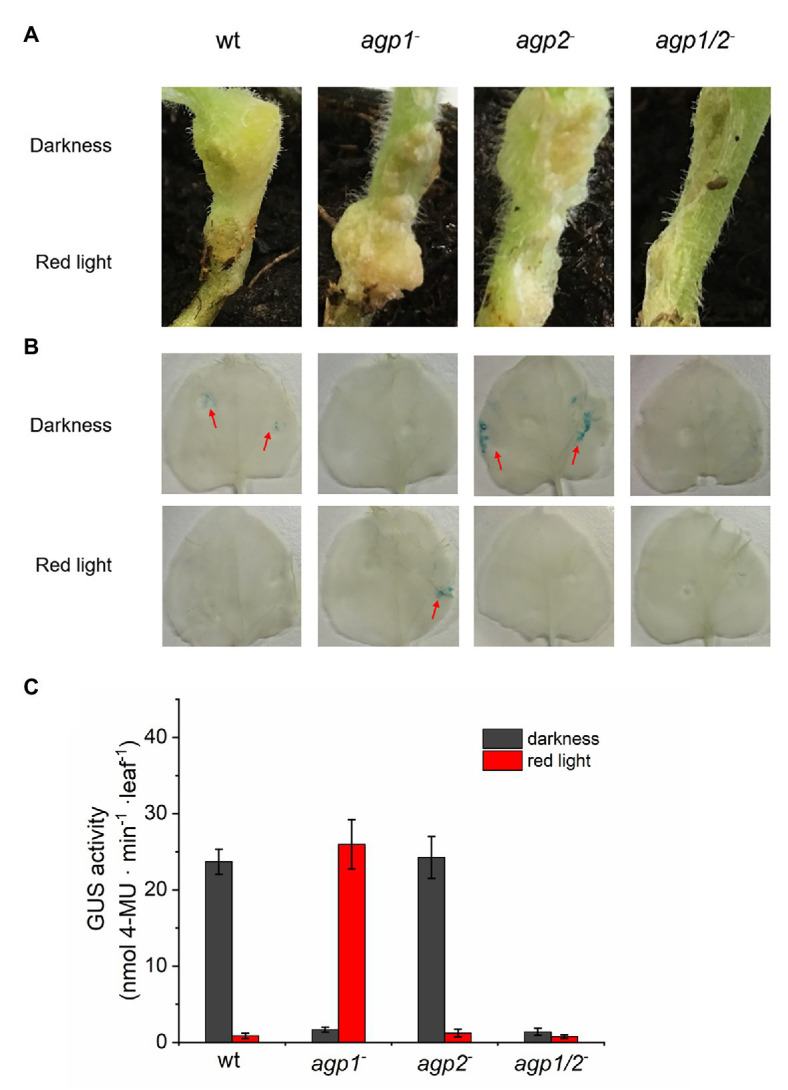
**(A)** Tumor induction in stems of *Nicotiana bentamiana*. Stems infected by *A. fabrum* wild-type (WT) and phytochrome mutants as indicated in the panels. During 1 day infection, the upper part of the stem was covered with aluminum and the entire plant placed in red light (1 μmol m^−2^ s^−1^). Stems with or without tumors were photographed after 6 weeks. **(B)** *A. fabrum* with pBIN-GUS plasmid was used for infection of *N. bentamiana* leaves, same strains as in **(A,C)** as in **(B)**, quantification by fluorescence based on MUG conversion (Xue et al., 2020).

Conjugation has many features in common with gene transfer into plants: in both cases, (i) single stranded DNA is transferred, (ii) there is a covalent link between a transfer protein and the adduct between DNA and the transfer protein is transmitted, and (iii) a type IV secretion system is involved in the transfer. We, therefore, investigated the gene transfer into plants again (Xue et al., 2020). In those experiments, tumor induction in *Arabidopsis* roots, tumor induction in *Nicotiana* stems, and DNA transfer into *Nicotiana* leaves were light controlled and dependent on phytochromes. In all cases, the effect was reduced in the wild type by red light and low in the agp1^−^/agp2^−^double mutant. Figure 8 shows examples for stem and leaf infection. So, clearly, two DNA transfer processes of *A. fabrum*, conjugation and plant infection, are under phytochrome control.

Plant infection has also been reported to be regulated by light *via* phytochrome in the plant pathogenic bacterium Xanthomonas campestris pv. Campestris (Bonomi et al., 2016). Light controlled infection of mammalian cells by Brucella species has been reported to be regulated by blue light photoreceptors with a LOV domain (Swartz et al., 2007). In these cases, infection is, however, not related to a DNA transfer, which is unique to the genus *Agrobacterium*.

We also found phytochrome effects on motility in *A. fabrum*. (for phytochrome effects on swarming of Pseudomonas syringae, see Wu et al., 2013). The effects on *A. fabrum* motility were difficult to analyze because broad pH variations of the medium and sophisticated data analyses were required to see effects. There was no evidence for a red light effect on motility in these experiments, but in certain pH conditions, motility was different between mutants and wild type (Xue et al., 2020). In case of cell growth, a comparable effect was found. There was no light regulation on cell growth (at least at ambient temperature), but a clear difference between wild type and mutants: the mutants grew faster.

The *A. fabrum* phytochrome effects can thus be divided into light dependent effects, such as plant infection and conjugation, and light independent effects, such as cell division or motility. The present general view of photoreceptor function is that action is induced only upon photoconversion. Such a clear separation between dark = inactive and light = active has long been discussed for plant phytochromes. If phytochrome would act in darkness, then a phytochrome mutant should have a phenotype in the dark, which is usually not the case (Dehesh et al., 1993). The results of *A. fabrum* show that these phytochromes can be active in darkness and in the light, a feature also found for fungal phytochromes (Yu et al., 2019).

A proteome study, in which dark and light grown wild type and the double knockout mutant were compared, showed that the abundance of many proteins differed between mutant and wild type, but only few differed between dark and light. This study confirms that phytochrome is sometimes active in darkness and in other cases requires light stimulation (Xue et al., 2020).

The dark activity reminds of autophosphorylation of the Agp1 histidine kinase, which is stronger in darkness than in the light. Such a dark/light pattern is found for kinase activity of other bacterial phytochromes as well. Note that autophosphorylation was found to be temperature dependent in an unexpected way (Njimona et al., 2014), as it decreased with increasing temperature. Hence, phytochrome effects were later analyzed at different temperatures (Bai et al., 2016). Phytochrome regulation at 25°C was always different from that at 37°C, and the results are in line with a temperature sensor function of phytochromes. For a clear understanding of the role of phytochrome in temperature sensing, the different steps in signal transduction must be unraveled.

Because both Agp1 and Agp2 act together in *A. fabrum*, we also investigated whether these proteins do interact physically. Indeed, with spectral assays, phosphorylation assays and FRET, we could demonstrate a weak interaction between both proteins (Xue et al., 2019). Because there is no evidence for transcriptional control by Agp1 and Agp2, we assume that also the regulation of conjugation and DNA transfer is mediated through protein interaction. Since the proteins are known that are involved in these processes, it will be interesting to follow up on interaction partners and to understand more details of the signal transduction cascade.

### Outlook

The phytochrome system of *A. fabrum* provides a model for studying the entire signal transduction, including early steps in photoreceptor photoconversion, protein conformational changes, and signal transmission *via* intermediate proteins to biological effects such as infection or conjugation. In the future, our group will continue with studies on TraA, which initiates the conjugation process, and VirD2, which initiates gene transfer to plants. Indirect evidence suggests that both could be directly controlled by Agp1 and Agp2.

## Author Contributions

All authors listed have made a substantial, direct and intellectual contribution to the work, and approved it for publication.

### Conflict of Interest

The authors declare that the research was conducted in the absence of any commercial or financial relationships that could be construed as a potential conflict of interest.
